# Neuronal hyperexcitability and impaired spatial coding in Alzheimer's disease mouse models

**DOI:** 10.1002/alz70855_097237

**Published:** 2025-12-23

**Authors:** Abid Hussaini, Gustavo A Rodriguez, Radha Raghuraman, Andrew Aoun, Oliver Shetler

**Affiliations:** ^1^ Burke Neurological Institute at Weill Cornell Medicine, White Plains, NY, USA; ^2^ Columbia University Irving Medical Center, New York, NY, USA

## Abstract

**Background:**

Alzheimer's disease (AD) represents a progressive neurodegenerative disorder characterized by complex pathophysiological mechanisms that fundamentally alter neural circuit dynamics and cognitive processing. The accumulation of amyloid‐beta (Aβ) peptides and associated neuroinflammatory processes progressively compromise key neural networks, particularly within critical memory‐associated cortical regions such as the medial entorhinal cortex (MEC) and lateral entorhinal cortex (LEC). These neuroanatomical structures serve pivotal roles in spatial navigation, memory encoding, and contextual information processing, rendering them vulnerable to early pathological changes in neurodegeneration.

**Method:**

To elucidate the intricate neurophysiological alterations accompanying AD pathogenesis, we employed in vivo electrophysiological approaches utilizing two distinct transgenic mouse models: App NL‐G‐F knock‐in (APP KI) and EC‐App/Tau mice. We incorporated single‐unit electrophysiology recordings, enabling high‐resolution tracking of neuronal ensemble activities within open field experimental paradigms. Methodological approaches included spatial information scoring, computational analyses employing Earth Mover's Distance (EMD) metrics, and spatial decoding techniques to interrogate subtle neuronal firing characteristics.

**Result:**

Neurophysiological analyses revealed profound alterations in neuronal ensemble behaviors across both cortical structures. In the medial entorhinal cortex, APP KI mice demonstrated significant reductions in spatial information encoding capabilities, manifesting as markedly decreased spatial information scores compared to age‐matched control populations. Notably, characteristic neuronal subtypes such as border cells and grid cells exhibited pronounced instabilities in firing preferences and spatial periodicity, suggesting fundamental disruptions in spatial representation mechanisms. Complementary investigations of the lateral entorhinal cortex unveiled hyperactive neuronal populations characterized by diminished information content, increased neuronal sparsity, and compromised firing precision for object and trace cell populations.

**Conclusion:**

We provide empirical evidence demonstrating that AD‐associated pathological processes induce neurophysiological disruptions within entorhinal cortical networks. The observed alterations in neuronal ensemble dynamics, characterized by compromised spatial and contextual encoding capabilities, offer critical insights into the mechanistic underpinnings of cognitive decline. These neurophysiological investigations illuminate the intricate relationship between molecular pathogenesis and circuit‐level dysfunction, suggesting potential mechanistic pathways through which Aβ accumulation progressively impairs neural information processing. The research underscores the importance of understanding nuanced neuronal network transformations as fundamental contributors to cognitive deterioration in AD, potentially facilitating future therapeutic interventions targeting early pathological mechanisms.

